# How do early-life adverse childhood experiences mediate the relationship between childhood socioeconomic conditions and adolescent health outcomes in the UK?

**DOI:** 10.1136/jech-2020-213817

**Published:** 2020-11-01

**Authors:** Viviane S Straatmann, Eric Lai, Catherine Law, Margaret Whitehead, Katrine Strandberg-Larsen, David Taylor-Robinson

**Affiliations:** 1 Department of Public Health, Policy and Systems, University of Liverpool, Liverpool, UK; 2 NVS, Karolinska Institutet, Stockholm Universitet, Aging Research Center, Stockholm, Sweden; 3 Population, Policy and Practice, Institute of Child Health, University College London, London, UK; 4 Department of Public Health, University of Copenhagen, Kobenhavn, Denmark

**Keywords:** Health inequalities, cohort studies, child health, public health

## Abstract

**Background:**

Both adverse childhood experiences (ACEs) and adverse childhood socioeconomic conditions (SECs) in early life are associated with poor outcomes across the life course. However, the complex interrelationships between childhood SECs and ACEs are unclear, as are the consequences for health outcomes beyond childhood. We therefore assessed the extent to which early-life ACEs mediate the relationship between SECs and socioemotional behavioural problems, cognitive disability and overweight/obesity in adolescence.

**Methods:**

We used longitudinal data from the UK Millennium Cohort Study (MSC). Outcomes assessed at age 14 were socioemotional behavioural problems, cognitive disability and overweight/obesity. SECs at birth were measured by maternal education. Potentially mediating ACEs measured up to 5 years were verbal and physical maltreatment, parental drug use, domestic violence, parental divorce, maternal mental illness and high frequency of parental alcohol use. We used counterfactual mediation analysis to assess the extent to which ACEs mediate the association between SECs at birth and behavioural, cognitive and physical outcomes at age 14, estimating total (TE), natural direct and indirect effects, and mediated proportions.

**Results:**

Children with disadvantaged SECs were more likely to have socioemotional behavioural problems (relative risk (RR) 3.85, 95% CI 2.48 to 5.97), cognitive disability (RR 3.87, 95% CI 2.33 to 6.43) and overweight/obesity (RR 1.61, 95% CI 1.32 to 1.95), compared to those with more advantaged SECs. Overall, 18% of the TE of SECs on socioemotional behavioural problems was mediated through all ACEs investigated. For cognitive disability and overweight/obese, the proportions mediated were 13% and 19%, respectively.

**Conclusion:**

ACEs measured up to age 5 years in the MCS explained about one-sixth of inequalities in adolescents behavioural, cognitive and physical outcomes.

## INTRODUCTION

The concept of ‘adverse childhood experiences’ (ACEs) has gained popularity as a way of framing the public health implications of a range of harmful childhood experiences.^1 2^ These typically include abuse, neglect and indicators of possible household dysfunction affecting children such as parental mental health problems and alcohol and drug misuse.^2^ Although the prevalence of ACEs varies on the basis of the definitions used, there is a clear association with a range of adverse health outcomes across the life course.^3^ Findings from a cross-sectional UK survey of people aged between 18 and 70 years showed that almost half of those surveyed reported at least one ACE, while 12% reported four or more ACEs.^4^

The preschool period is a crucial stage of development that influences children’s subsequent development and health outcomes.^5^ Early childhood adversity in the preschool period influences lifelong health and is more common in children growing up in disadvantaged socioeconomic conditions (SECs).^6–8^ Our recent study, for example, has shown that socioeconomic inequalities in an adolescent mental health outcome can be partially explained by perinatal, individual child, family, peer relation and neighbourhood-level factors up to age 3.^9^ Some of these early-life risk factors (e.g. parental mental health and alcohol misuse, and bullied children) may be framed as ACEs,^9^ and reinforce the importance of these detrimental experiences in the early years^7^ and their potential role in mediating socioeconomic inequalities in health outcomes in adolescence.

**Figure 1:**
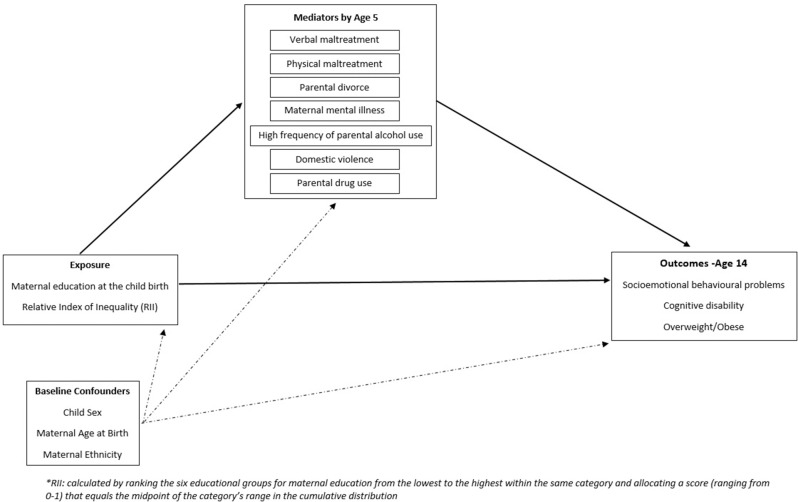
Direct acyclic graph of the natural direct effect of the exposure to outcomes at age 14, and the natural indirect effect throughout mediators by the age 5. Model adjusted for potential baseline confounders.

Several studies have highlighted the need to better understand the interrelationships between ACEs and childhood SECs.^6 10 11^ A recent systematic review showed that there is a clear relationship between disadvantaged childhood SECs and increased risk of ACEs.^6^ While we know that both adverse SECs and ACEs are harmful to health,^3 12 13^ from a causal pathway perspective, ACEs may be conceptualised as being mediators on the causal pathway from disadvantaged SECs to adverse outcomes. For example, as outlined in our logic model in figure 1, previous studies show that children living in disadvantaged SECs are more likely to be exposed to ‘toxic stress’ such as parental mental health problems, parental drug and alcohol misuse, and other family dysfunctions, which may lead to subsequent adverse health outcomes later in life.^9 11 14^

Furthermore, there has been a conflation of socioeconomic factors and ACEs, in some studies, which makes it difficult to differentiate these factors in relation to their contribution to the pathways to poorer health.^6^ Some investigations have included measures of SECs such as economic hardship, poverty and deprivation within their definition of ACEs measurement.^15 16^ By contrast, other authors have adjusted for SECs in order to assess independent associations between ACEs and later health outcomes.^4 11 17–20^ We have previously raised concerns that this might lead to the importance of SECs being overlooked when considering policy responses to ACEs.^10^

From a health inequality policy perspective, it is important to better understand the complex interrelationships of SECs and ACEs, and specifically, the extent to which ACEs mediate socioeconomic inequalities in later health outcomes, in order to develop appropriate public health strategies.^6 21 22^ This study, therefore, aims to explore the social patterning of ACEs measured in the preschool period in the latest UK birth cohort, and the extent to which these adversities mediate causal pathways between childhood SECs and three important outcomes which represent mental, cognitive and physical aspects of adolescent health (i.e. socioemotional behavioural problems, cognitive disability and overweight/obesity).

## METHODS

We conducted a causal mediation analysis using the Millennium Cohort Study (MCS). MCS is a large nationally representative cohort study of 18 818 children born in the UK between 2000 and 2002 and followed up at six intervals (9 months and ages 3, 5, 7, 11 and 14). To address our research questions, we used data from baseline (9 months), ages 3, 5 and 14 years. The main exposure was assessed at baseline (9 months), mediators in respective preschool ages (ages 3 and 5) and outcomes in the latest sweep available in the MCS (age 14). Survey interviews were carried out in the home with the main respondent (99% mother). The study oversampled children living in disadvantaged areas and in those with high proportions of ethnic minority groups by means of a stratified cluster sampling design.

### Measures

#### Health outcomes: behavioural, cognitive and physical

We investigated outcomes at 14 years, captured in the latest MCS data sweep. Following our previous study,^23^ and recognising the impact of ACEs on a broad range of health and developmental outcomes,^11^ we have used three outcomes to capture aspects of mental and physical health, and cognitive ability.

Socioemotional behaviour was assessed with the Strengths and Difficulties Questionnaire (SDQ—maternal reported).^24^ The SDQ is a 25-item measure that asks parents to rate their child’s behaviour over the previous 6 months using five subscales, each with five items: peer problems, conduct disorders, hyperactivity, emotional problems and prosocial behaviour. We used the total difficulties score (which excludes the prosocial behaviour items) using validated cut-offs used in previous studies^25 26^ for which a score of 0–16 indicates ‘normal to borderline behaviour’ and 17–40 indicates ‘socioemotional behavioural problems’.^24^ Cognitive ability was assessed through the word activity test which measures knowledge of vocabulary.^27^ The adolescents received 20 different words in English and five possible synonyms for each and were asked to match each word to its correct synonym. We applied a widely used cut-off score^23 28 29^ of −1.25 standard deviation (SD) below the normed mean score for the sample to define children as having vocabulary/cognitive disabilities. Overweight/obese was derived from the body mass index, using the age and sex-specific International Obesity Task Force cut-offs.^30^

### Measurement of SECs

The level of maternal education at birth was our primary exposure of interest, used as a measure of childhood SECs. The highest qualification attained by the mother was established by questionnaire at the first sweep ((1) ‘Degree plus=higher degree or first-degree qualifications’; (2) ‘Diploma=in higher education’; (3) ‘A-levels’; (4) ‘General Certificate of Secondary Education (GCSE) grades A–C’; (5) ‘GCSE grades D–G’; (6) ‘None of these qualifications’). Maternal educational level has been used in previous studies exploring inequalities in child health^31 32^ and represents a more stable measure of SECs as compared to income, which could fluctuate at times. It also encompasses a range of non-economic social attributes such as general and health-related knowledge, literacy, problem-solving skills and prestige.^12^

### ACEs in the preschool period

Based on the original classification of ACEs presented by Felitti and colleagues,^1^ a block of seven parental reported potentially mediating ACEs experienced by the child up to 5 years were captured in the MCS: physical maltreatment, verbal maltreatment; parental drug use, high frequency of parental alcohol use, domestic violence, parental divorce and maternal mental illness. These ACEs are commonly used in other studies, which can facilitate comparisons.^3 6 11^ The full details of coding of the potential mediators are provided in online supplemental material S2 and online supplemental material S3. In the mediation analysis, we used the naturally occurring coding of the mediators in the MCS (multicategory variables) to maximise power to capture mediation through our ACEs variables. Each ACEs variable was included in the model in its original format.

10.1136/jech-2020-213817.supp1 Supplementary data

### Analysis

First, we estimated the prevalence of the health outcomes at age 14. We then assessed the distribution of our health outcomes and ACEs according to the level of maternal education at birth using the χ^2^ test. For descriptive purposes, we operationalised ACEs variables by combining categories that distinguished the most adverse scenarios (binary variables) (see description in online supplemental material S2). According to this operationalisation, we presented the frequency of children that have 1, 2, 3, 4 or more ACEs by level of maternal education at birth.

Second, we undertook formal mediation analysis using the counterfactual framework, an approach that provides a clear and coherent framework to think about a variety of important concepts related to causation,^33^ to assess the amount of social inequality in health outcomes at age 14 attributable to ACEs experienced up to age 5, while adjusting for baseline demographic factors (child sex, maternal ethnicity and maternal age at birth). In the mediation analysis, we used the naturally occurring coding of the mediators in the MCS (multicategory variables), shown in online supplemental material S3, to maximise power to capture mediation through the investigated ACEs. We scaled the education measure in order to derive a measure of the relative index of inequality (RII). The RII compares the risk of poor health outcomes between children of lowest and highest SECs, taking into account the distribution of education level in the study population by ranking the maternal education groups from lowest to highest and allocating a score (ranging from 0 to 1) that represents the midpoint of the category’s range in the cumulative distribution.^34^ We used this scaled measure in our regression models to derive the RII, which summarises the relative risk (RR) across the socioeconomic gradient in the population.^34 35^ We estimated the RR and 95% CI for the natural direct effect (NDE), natural indirect effect (NIE) and total effect (TE) (formulas are shown in online supplemental material S1) for the direct acyclic graph outlined in figure 1, considering all ACEs. We calculated the proportion mediated and 95% CI applying the formula: (RR^NDE^*(RR^NIE^-1))/(RR^NDE^*RR^NIE−1^).^36^ Analysis was conducted in Stata/SE V.15 (Stata Corporation) and in *medflex* package of R software^37^ R V.3.4.4.

### Robustness tests and additional analysis

To explore exposure–mediator interaction, we repeated the analysis allowing for all two-way interactions between maternal education and the mediators in the model and used the Akaike information criterion (AIC) to compare model fit. We repeated the counterfactual mediation analysis using equivalised family income as an alternative measure of childhood SECs. We also repeated our analysis without the alcohol variable, since our variable only captures the frequency of parental alcohol consumption, and not the volume. It is thus possible that our variable may not reflect an adverse experience for the child. We also explored the mediating ACEs effect in socioeconomic inequalities in other health risk behaviour outcomes such as smoking, alcohol and cannabis experimentation at age 14. Finally, we repeated the main analysis with multiple imputed data sets.

## RESULTS

There were respectively 11 169, 10 645 and 10 825 children who participated in the first and the latest sweeps of MCS and who had data on socioemotional behavioural problems, cognitive disability and overweight/obese at age 14. Around two-thirds (N=6499 (socioemotional behavioural problems); 5393 (cognition); 6306 (overweight/obese)) had data on the exposure, outcomes, mediators and confounders of interest, that is, the complete case population to each outcome.

At age 14, 8.7% (95% CI 7.9% to 9.7%) of children had socioemotional behavioural problems, 6.0% (95% CI 5.2% to 7.0%) had cognitive disabilities and 24.6% (95% CI 23.3% to 25.9%) were overweight/obese, with a clear social gradient in all outcomes (table 1). In our study, 50% of children had experienced one or more ACEs, 16.4% two or more, 5.4% three or more and 1.4% four or more ACEs. According to the binary operationalisation of our ACEs variables, the most prevalent ACEs was verbal maltreatment at age 5 (36.5%), followed by high frequency of parental alcohol consumption at age 5 (8.2%). There were also significant social gradients evident in many ACEs, apart from verbal and physical maltreatment, and use of drugs. All socially patterned ACEs were more common in children growing up in more disadvantaged circumstances, apart from high frequency of alcohol consumption, which was more common in more socially advantaged families (table 1). The social patterning of ACEs using the natural categories coding available in the MCS (multicategories) can be found in online supplemental material S3.

**Table 1:** Health outcomes at age 14 and early adverse childhood experiences by maternal educational level at birth

	Total	Degree plus	Diploma	A-level	GCSE A−C	GCSE D–G	None	
Maternal educational level at birth	%	%	%	%	%	%	%	P value*
Outcomes								
Social emotional behavioural problems	8.7	3.5	7.0	6.4	8.4	15.3	15.4	*<0.001*
Cognitive disability	6.0	2.5	4.9	4.2	6.5	9.1	9.9	*<0.001*
Overweight/obese	24.6	16.7	23.9	21.0	26.5	30.0	30.8	*<0.001*
ACEs**								
Verbal maltreatment (age 5)	36.5	34.1	37.5	38.6	38.6	35.6	32.9	0.067
Physical maltreatment (age 5)	1.4	0.8	1.0	1.2	1.5	1.3	2.2	0.309
Parental divorce (age 3)	3.4	1.0	3.6	3.1	3.9	3.7	5.5	*<0.001*
Parental divorce (age 5)	2.1	4.7	4.7	3.9	4.3	4.9	8.7	*<0.001*
Maternal mental illness (age 3)	2.3	0.5	1.1	1.2	2.5	3.9	5.0	*<0.001*
Maternal mental illness (age 5)	2.4	0.6	1.9	1.1	2.3	4.7	5.1	*<0.001*
High frequency alcohol use (age 3)	7.3	13.3	9.7	8.9	5.2	4.4	3.3	*<0.001*
High frequency alcohol use (age 5)	8.2	13.9	9.7	7.9	7.2	5.6	3.4	*<0.001*
Domestic violence (age 3)	3.3	2.6	4.5	2.6	2.9	4.3	5.1	*0.018*
Domestic violence (age 5)	4.1	3.0	3.1	3.6	4.1	5.1	6.2	*0.048*
Use of drugs	0.8	0.5	0.9	0.4	0.7	0.8	1.6	0.317
≥1 ACEs	50.8	49.2	53.7	52.6	50.3	51.5	50.5	0.660
≥2 ACEs	16.4	16.1	16.4	14.4	15.2	18.4	19.9	0.198
≥3 ACEs	5.4	5.3	5.7	3.5	6.1	3.8	6.1	0.185
≥4 ACEs	1.4	1.1	1.7	0.9	1.6	0.5	2.1	0.210

*Data in italic on significance level of <0.05.

**ACEs were dichotomised as outlined in online supplemental material S2 for descriptive analysis.

ACEs, adverse childhood experiences; GCSE, General Certificate of Secondary Education.

**Figure 2:**
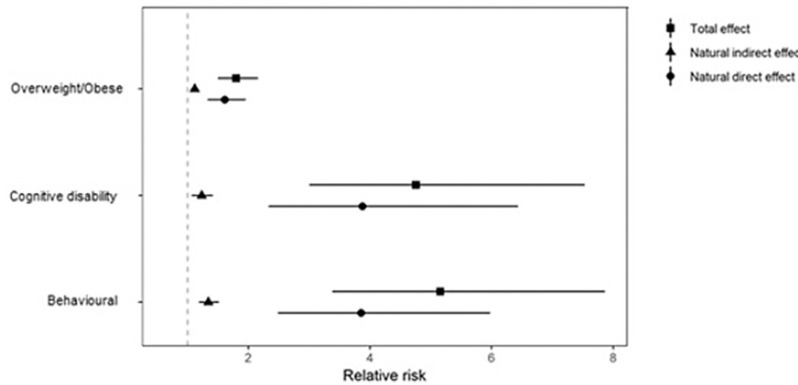
Counterfactual mediation analysis. Relative risk (95% CI) of natural direct effect (95% CI), natural indirect effect (95% CI) and total effect (95% CI) for relative index of inequality by all adverse childhood experiences for behavioural, cognitive and overweight/obese outcomes at age 14.

The results of the counterfactual mediation analysis are illustrated in figure 2. Taking socioemotional behavioural problems as an example, the TE was RR 5.16 (95% CI 3.37 to 7.86). The NDE (RR 3.85, 95% CI 2.48 to 5.97) is the increase in socioemotional behavioural problem risk comparing low to high SECs that we would observe if the levels of ACEs were set to those experienced by children at the top end of the SEC hierarchy; and the NIE is the increased risk of socioemotional behavioural problems we would see if the SECs were fixed at top of the SEC hierarchy, but the ACEs mediators were fixed at those that would naturally occur at low SECs (RR 1.33, 95% CI 1.18 to 1.51), compared to if they remained at the high SEC levels.

There was a significant indirect effect of SECs on our outcomes of interest via ACEs experienced up to 5 years of age, indicating statistically significant mediation. Respectively, 18% (95% CI 9.9% to 28.1%), 13% (95% CI 3.7% to 26.2%) and 19% (95% CI 8.7 to 32.7%) of the TE of SECs on risk of socioemotional behavioural problems, cognitive disability and overweight/obese at age 14 was mediated through adversities measured in the MCS by the age five (details in online supplemental material S4).

### Robustness tests and additional analyses

For the counterfactual mediation analysis, a model that included all exposure-mediator interactions had a worse fit (i.e. higher values) based on the AIC (results are not shown). Conclusions were similar when we repeated the analysis using RII based on family income as the main SECs measure, instead of maternal education (online supplemental material S5). Regarding the analysis excluding the mediating variable of frequency of parental alcohol consumption, we found an attenuation on the proportion mediated by ACEs for socioemotional behaviour problems (18%, 95% CI 9.9% to 28.1% vs 11.8%, 95% CI 6.4% to 18.7%) and cognitive disability (13%, 95% CI 3.7% to 26.2% vs 7.5%, 95% CI 0.9% to 16.5%) outcomes at age 14, but the results were similar. There was no mediation by ACEs for overweight/obese when removing the alcohol variable (NIE RR 1.03, 95% CI 0.99 to 1.07) (online supplemental material S6). There was no mediating effect of ACEs experienced up to 5 years of age for the other health risk behaviours at age 14 (details in online supplemental material S7). Analysis using multiple imputed data sets (online supplemental material S8) showed similar patterns of the main analysis.

## DISCUSSION

Using nationally representative data from the UK, we show that most ACEs captured in the MCS by the age of 5 years, with the exception of parental alcohol consumption, are more common in more disadvantaged children, and they explain about one-sixth of socioeconomic inequalities in socioemotional behavioural problems (17%), cognitive ability (13%) and overweight/obesity (18%) at age 14.

The stark inequalities in adolescent health outcomes illustrated in our study corroborate other studies.^38–41^ Ours is one of the first studies quantifying ACEs in the preschool period using a rich cohort data. In our study, the most prevalent ACEs were verbal maltreatment and high frequency of parental alcohol consumption at age 5; 1.4% of children had experienced ≥4 ACEs. Bellis and colleagues^4^ observed a prevalence of 12.3% for ≥4 ACEs in a retrospective cross-sectional survey of 1500 residents and 67 substance users aged 18–70 years in a relatively deprived and ethnically diverse UK population. The prevalence of ACEs in our study is not directly comparable with other studies, which have focused on self-report data in adulthood, since we used estimates of ACEs restricted to the preschool period (up to 5 years of age). Moreover, these differences in prevalence may also be explained by different operationalisation of ACEs variables (i.e. composite measures of ACEs vs single variables of ACEs).

Our study presents a causally informed interpretation of the degree to which ACEs might be responsible for the generation of socioeconomic inequalities in health. We show that ACEs mediate a small but significant proportion of the effect of SECs on the health outcomes chosen in our study. Turning to our logic model in figure 1, there are now data from many studies showing that children growing up in adverse SECs are more likely to experience ACEs such as parental mental health problems.^6^ Walsh and colleagues^6^ systematically reviewed the relationship between childhood SECs and ACEs, and found that there is a clear relationship between disadvantaged SECs in childhood and risk of experiencing ACEs and maltreatment. This relationship appears robust across countries, measures of SECs and the age at which adversity is measured.^6^ The longitudinal nature of many of the studies systematically reviewed by Walsh *et al^6^* supports a causal association between SECs in childhood and ACEs/maltreatment.

We also know that there are stark inequalities in most child health outcomes^13^ and that childhood SECs are a fundamental cause of health inequalities, important in explaining variation in outcomes across the life course.^6^ Returning to our logic model in figure 1, several studies have suggested a causal impact of ACEs on health across the life course with effects that persist even after adjustment for measures of SECs.^4 17–20^ The question then follows as to how ACEs mediate the association between SECs and adverse outcomes in adolescence. To our knowledge, this is the first study to address this question. A recent study using data from the Avon Longitudinal Study of Parents and Children (ALSPAC) showed that most of the individual ACEs were associated with lower educational attainment, and there was attenuation of many of the ACEs associations with health and education outcomes after adjusting for SECs, particularly for education. While the study did not undertake formal mediation analysis, these results also suggest a role for ACEs in mediating child health inequalities.^11^ Another recent publication also using the ALSPAC cohort investigated clustering of ACEs and whether this is predicted by poverty. Poverty was strongly associated with all adversity clusters and more strongly related to the multiple adversity cluster, leading the authors to conclude that poverty alleviation may be a critical element of ACEs reduction.^42^

### Strengths and limitations

A key strength of our study is the use of a large, contemporary UK cohort, which measures a wide range of information, which allowed us to explore a range of ACEs experienced in the first 5 years of life. This study adds to the literature by being the first to formally test the mediating role of ACEs lived through a key stage of life (i.e. first infancy) on social inequalities in adolescent outcomes using counterfactual methods. The use of validated measures of adolescent mental health, cognitive performance and overweight/obesity is also a strength of our study. Another strength is that we repeated our analysis and showed similar results using different dimensions of SECs at baseline (main analysis: maternal education and additional analysis: income). Future analyses could be repeated using neighbourhood measures of SECs at birth.

The use of parent-reported data on ACEs is a potential limitation that may increase chances of underreporting and underestimating the mediation effect of ACEs on our findings. In this study, we were interested in quantifying the impact of adversities experienced in the preschool period; thus, our findings may be considered as the minimum impact of ACEs on inequalities in adolescents’ outcomes. Another limitation is that the MCS, as with many other studies, does not have data on all events considered as ACEs (e.g. sexual abuse, incarceration of adults in the household, etc). MCS questions on parental alcohol consumption at ages 3 and 5 only evaluated frequency without quantifying the volume, and we therefore undertook an additional analysis removing this variable from our mediating block.

Sampling and response weights were used for descriptive analysis to account for the sampling design and attrition to age 14; however, these cannot account for item missingness. In this analysis, the sample was large, and the internal associations, which were the targets of inference within the sample population, are likely to be valid. Although we used modern methods for causal mediation analysis, and adjusted for a range of potential confounders, the assumption of complete adjustment of confounding is still required for causal interpretation of our estimates. Future research may focus on analysing the cumulative mediating role of ACEs up to age 18 and the importance of experiences at specific stages of childhood (i.e. school age) for health inequalities. Other outcomes could also be assessed such as physical health conditions and school attendance.

### Policy, practice and research implications

The increased risk of socioemotional behavioural problems, cognitive disability and overweight/obesity in adolescents growing up in disadvantaged SECs in the UK is only partly explained by ACEs measured up to 5 years of age. Our results suggest that public health policies to address socioeconomic inequalities in adolescent health outcomes in the UK should focus on continued actions on social determinants of health. At the same time, modifiable consequences of socioeconomic disadvantages leading to increased exposure to ACEs should be addressed. Appropriate intervention is likely to vary widely depending on the specific exposure. Taking the example of parental mental health problems as an ACE, this might involve peer support delivered in children’s centres in disadvantaged areas to help prevent mental health problems in parents occurring, coupled with early and effective services for families if there are mental health problems in any family members.

In addition, further research is needed to understand the myriad pathways by which socioeconomic disadvantages cause poor health outcomes, and to understand the dynamics of how childhood adversities interact to generate poor health outcomes and their consequences, with a focus on using this understanding to effect change.

**What is already known on this subject**

- Adverse childhood experiences (ACEs) are highly socially patterned and associated with increased risk of negative outcomes throughout the life course, but less consideration has been given to their role in generating health inequalities.

**What this study adds**

- About a sixth of the increased risk of socioemotional behavioural problems, cognitive disability and overweight/obesity in adolescents growing up in disadvantaged socioeconomic circumstances in the UK is explained by adversities experienced up to 5 years of age.

**Policy implications**

- Public health policies to improve adolescent health outcomes in the UK should focus on continued action on the social determinants of health. At the same time, modifiable consequences of socioeconomic disadvantage should be addressed, with appropriate prevention and support to mitigate the impact of adverse childhood experiences.
