# Supplementary material for: How do early-life adverse childhood experiences mediate the relationship between childhood socioeconomic conditions and adolescent health outcomes in the UK?
Source: J Epidemiol Community Health. 2020 Nov 1;74(11):969–75. doi: 10.1136/jech-2020-213817 (PMC7576580; doi:10.1136/jech-2020-213817)
Supplement: Supplementary data [file jech-2020-213817s001.pdf]

Online only supplementary material

How do adverse childhood experiences mediate the relationship between childhood socio-economic conditions and health outcomes in the UK?

Viviane S Straatmann; Eric TC Lai; Catherine Law; Margaret Whitehead; Katrine Strandberg-Larsen, David Taylor-Robinson.

This data supplement contains additional information on the methods employed in the study. In addition, further plots and results are presented.

Supplementary material S1. Formulas and types of confounding

Natural Indirect Effect (NIE): g (E (Y (aM (a))-Y (aM (a*))))
Total Effect (TE): g (E (Y (a)-Y (a*)))

The four types of confounding are: (1) confounding of the exposure-outcome relationship; (2) confounding of the mediator-outcome relationship; (3) confounding of the exposure-mediator association; and (4) mediator-outcome confounders also affected by the exposure. For controlled direct effect, assumptions (1) and (2) are required. For the identification of natural direct and indirect effects, assumptions (3) and (4) are also needed.

Supplementary material S2- Text box: Description of early adverse childhood experiences at ages 3 and 5

- **Verbal maltreatment (Age 5) -** the main responder was questioned about *'How often shouts at child when naughty? Daily, often [about once a week or more], sometimes [once a month], rarely or never'*.
  Dichotomized: daily, often *versus* sometimes/rarely/never [Ref]

- **Physical maltreatment (Age 5) -** the main responder was questioned about *'How often smacks the child when naughty? Daily, often [about once a week or more], sometimes [once a month], rarely or never'*.
  Dichotomized: daily, often *versus* sometimes/rarely/never [Ref]

- **Parental divorce (Ages 3 and 5)[1] -** the main responder was asked about marital status to identify occurrence of divorce or legal separation (*'Divorced, legally separated, 1st marriage, remarried, 2nd or later married, single, never married or widowed'*).
  Dichotomized: divorced/legally separate *versus* single/widowed/married [Ref]

- **Maternal metal illness (Ages 3 and 5)[1] -** Kessler 6 (K6)[¥] scale was used to assess maternal mental health in the last month asking the responders how often they felt depressed, hopeless, restless or fidgety, worthless, or that everything was an effort. Respondents answered on a five-point scale from 1 (all the time) to 5 (none of the time). We reversed and rescaled all items from 0 to 4 for analysis purposes, so that high scores indicate high levels of psychological distress. We used validated cut offs for severe mental illness [*'yes (scores >=13) /no'*]

- **High frequency of parental alcohol use (Ages 3 and 5)[1] -** the main responder responded a question about the usual frequency of alcohol consumption (*'Every day, 5-6 times per week, 3-4 times per week, 1-2 per week, 1-2 per month, less than once a month or never'*).
  Dichotomized: every day and 5-6 times per week *versus* 3-4 per week/1-2 per week/1-2 per month/never [Ref]

- **Domestic violence (Ages 3 and 5)[1] -** the main responder was asked about the use of force by the partner in relationship (*'Yes, no or do not want to answer'*
  Dichotomized: yes/do not want to answer *versus* no [Ref]

- **Use of drug (Age 5) -** the main responder was asked about the use of recreational drugs in past 12 months (*'regularly, occasionally, cannot define or never'*).
  Dichotomized: regularly *versus* occasionally, cannot define and never [Ref]

[1]Information measured at age 3 and 5 were treated as two independent variables in the models

[¥] Kessler RC, Andrews G, Colpe LJ, Hiripi E, Mroczek DK, Normand SLT et al. Short screening scales to monitor population prevalences and trends in non-specific psychological distress. Psychological Medicine 2002; 32(6), 959–976.

Supplementary material S3. Health outcomes at age 14 and potentially mediating variables by maternal educational level at birth

Maternal educational level at birth	Degree Plus	Diploma	A level	GCSE A-C	GCSE D-G	None	Total	p-value
	%	%	%	%	%	%	%	
**ACES**								
**Verbal maltreatment**								0.001
Daily	2.7	4.7	3.8	5.2	6.2	6.1	4.7	
Often	31.4	32.7	34.7	33.4	29.4	26.8	31.8	
Sometimes	39.4	38.5	39.3	37.0	39.0	32.3	37.5	
Rarely	24.1	21.7	20.7	21.9	24.3	32.6	23.8	
**Physical maltreatment**								<0.001
Daily	0.03	0	0.2	0.01	0.3	0.4	0.10	
Often	0.8	1.0	0.9	1.5	1.0	1.9	1.3	
Sometimes	7.9	10.9	11.4	10.8	9.0	11.0	10.1	
Rarely	36.7	44.5	42.1	49.3	48.5	44.1	44.8	
**Parental divorce age 3**								<0.001
Legally separate	0.3	0.8	0.3	1.1	1.6	2.0	1.0	
Remarried	5.1	5.5	6.3	8.3	7.4	7.7	7.0	
Single	8.8	9.8	13.6	22.2	28.5	36.1	19.9	
Divorced	0.7	2.8	2.8	2.7	2.6	3.5	2.4	
Widowed	0	0	0	0	0	0.04	0.004	
**Parental divorce age 5**								<0.001
Legally separate	0.9	0.6	1.0	1.4	1.6	2.3	1.3	
Remarried	5.7	5.7	7.2	9.0	7.7	6.2	7.3	
Single	7.2	8.2	12.3	18.6	26.9	35.5	17.2	
Divorced	1.1	4.1	2.9	3.0	3.2	6.4	3.2	
Widowed	0	0	0	0	0.04	0	0.005	
**Maternal mental illness age 3**								<0.001
Yes	0.5	1.1	1.2	2.5	3.9	5.0	2.3	
**Maternal mental illness age 5**								<0.001
Yes	0.6	1.9	1.1	2.3	4.7	5.1	2.4	
**Frequency of parental alcohol use age 3**								<0.001
Every day	4.5	4.8	4.1	2.6	3.2	2.3	3.4	

5-6 p/ week	8.8	4.8	4.8	2.6	1.2	1.0	3.9	
3-4 p/ week	20.8	15.8	12.2	11.1	7.6	4.6	12.4	
1-2 p/ week	31.1	31.5	32.0	28.0	21.1	20.7	27.7	
1-2 p/ month	13.2	18.5	19.3	20.5	21.2	21.4	18.9	
< once a month	12.1	14.0	16.2	21.7	29.3	23.5	19.5	
**Frequency of parental alcohol use age 5**								<0.001
Every day	5.3	4.7	3.5	3.2	3.7	1.8	3.7	
5-6 p/ week	8.6	5.0	4.5	3.9	2.0	1.6	4.5	
3-4 p/ week	23.1	15.7	15.3	11.7	8.5	4.5	13.5	
1-2 p/ week	28.0	31.3	25.7	26.7	23.3	23.2	26.5	
1-2 p/ month	15.1	16.6	20.5	19.8	22.7	17.3	18.6	
< once a month	10.5	13.7	20.0	21.1	24.7	25.0	19.0	
**Use of drugs**								0.03
Occasionally	1.6	2.8	1.9	3.8	5.1	3.0	3.1	
Regularly	0.5	0.9	0.4	0.7	0.8	1.6	0.8	
Cannot say	0.6	1.0	0.9	0.6	1.4	1.1	0.8	
**Domestic violence age 3**								<0.001
Do not want to answer	0.9	2.0	2.2	3.8	4.3	6.4	3.4	
Yes	2.9	4.8	4.3	4.2	5.2	6.2	4.5	
**Domestic violence age 5**								0.06
Do not want to answer	1.9	1.6	3.2	2.8	3.1	4.4	2.9	
Yes	3.1	3.8	3.5	4.1	4.9	5.2	4.1	

Reference categories were omitted

Supplementary material S4. Natural Direct Effect (NDE), Natural Indirect Effect (NIE), Total Effect (TE) and proportion mediated for Relative Index of Inequality (RII) by ACEs for adolescents behavioural, cognitive and overweight/obese at age 14

Health outcomes	Effect RR (95% CI)			Proportion mediated
	NDE	NIE	TE	% (95% CI)
**Social emotional behavioural problems**	*3.85 (2.48, 5.97)*	*1.33 (1.18, 1.51)*	*5.16 (3.37, 7.86)*	*17.8 (9.9, 28.1)*
**Cognitive disability**	*3.87 (2.33, 6.43)*	*1.22 (1.06, 1.41)*	*4.75 (3.00, 7.53)*	*13.1 (3.7, 26.2)*
**Overweight/Obese**	*1.61 (1.32, 1.95)*	*1.11 (1.05, 1.17)*	*1.79 (1.49, 2.15)*	*18.6 (8.7, 32.7)*

NDE: Natural Direct Effect; NIE: Natural Indirect Effect; TE: Total Effect; RII based on maternal education

Supplementary material S5. Natural Direct Effect (NDE), Natural Indirect Effect (NIE), Total Effect (TE) and proportion mediated for Relative Index of Inequality (RII) (using income as alternative exposure) by ACEs for adolescents behavioural, cognitive and overweight/obese at age 14

Health outcomes	Effect RR (95% CI)			Proportion mediated
	NDE	NIE	TE	% (95% CI)
**Social emotional behavioural problems**	3.84 (2.27, 6.53)	1.27 (1.11, 1.52)	4.86 (3.00, 7.87)	14.9 (3.1, 30.4)
**Cognitive disability**	3.80 (2.27, 6.40)	1.20 (1.01, 1.44)	4.57 (2.90, 7.21)	12.2 (0.6, 27.5)
**Overweight/Obese**	1.65 (1.35, 2.00)	1.12 (1.04, 1.20)	1.85 (1.54, 2.22)	18.3 (6.7, 34.6)

NDE: Natural Direct Effect; NIE: Natural Indirect Effect; TE: Total Effect

Supplementary material S6. Natural Direct Effect (NDE), Natural Indirect Effect (NIE), Total Effect (TE) and proportion mediated for Relative Index of Inequality (RII) by ACEs for adolescents behavioural, cognitive and overweight/obese at age 14- excluding the mediating variable of frequency of parental consumption of alcohol

Health outcomes	Effect RR (95% CI)			Proportion mediated
	NDE	NIE	TE	% (95% CI)
**Social emotional behavioural problems**	4.32 (2.81, 6.61)	1.22 (1.12, 1.33)	5.28 (3.43, 8.01)	11.8 (6.4, 18.7)
**Cognitive disability**	4.30 (2.62, 7.06)	1.13 (1.01, 1.25)	4.84 (3.05, 7.70)	7.5 (0.9, 16.5)
**Overweight/Obese**	1.77 (1.47, 2.15)	1.03 (0.99, 1.07)	1.82 (1.52, 2.19)	-

NDE: Natural Direct Effect; NIE: Natural Indirect Effect; TE: Total Effect; RII based on maternal education

Supplementary material S7. Natural Direct Effect (NDE), Natural Indirect Effect (NIE) and Total Effect (TE) for Relative Index of Inequality (RII) by ACEs alcohol, smoking and cannabis experimentation at age 14

Health outcomes	Effect RR (95% CI)		
	NDE	NIE	TE
**Alcohol experimentation**	1.22 (1.07, 1.38)	0.91 (0.87, 0.95)	1.11 (0.99, 1.25)
**Smoking**	1.91 (1.41, 2.59)	1.04 (0.95, 1.14)	2.00 (1.50, 2.66)
**Cannabis experimentation**	1.11 (0.61, 2.01)	1.22 (0.99, 1.48)	1.35 (0.77, 2.38)

NDE: Natural Direct Effect; NIE: Natural Indirect Effect; TE: Total Effect; RII based on maternal education

Supplementary material S8. Multiple imputation**:** Natural Direct Effect (NDE), Natural Indirect Effect (NIE), Total Effect (TE) for Relative Index of Inequality (RII) by ACEs for adolescents behavioural, cognitive and overweight/obese at age 14

**Health outcomes**	**Effect RR (95% CI)**		
	**NDE**	**NIE**	**TE**
**Social emotional behavioural problems**	3.75 (2.57, 5.47)	1.43 (1.26, 1.62)	5.40 (3.77, 7.74)
**Cognitive disability**	4.07 (2.63, 6.29)	1.26 (1.11, 1.43)	5.16 (3.41, 7.78)
**Overweight/Obese**	2.23 (1.80, 2.76)	1.18 (1.10, 1.25)	2.61 (2.13, 3.22)

NDE: Natural Direct Effect; NIE: Natural Indirect Effect; TE: Total Effect; RII based on maternal education
